# METHODOLOGICAL CONCERNS FROM A MIXED-METHODS STUDY OF FRIENDSHIP IN PERSONS WITH DEMENTIA LIVING IN LTC

**DOI:** 10.1093/geroni/igad104.0986

**Published:** 2023-12-21

**Authors:** Pamela Saunders, Kate de Mederios

**Affiliations:** Georgetown University, Washington, District of Columbia, United States; Miami University, Oxford, Ohio, United States

## Abstract

This paper will examine the methodological issues that arose as part of the Friendship Study (de Mederios, Saunders, Doyle, Mosby, 2012). These issues range from tracking to integrating data as well as ethical considerations of multiple participants living with dementia and their legally authorized representatives. The Friendship Study investigated ‘friendships’ in people with dementia living in long-term care. We used a mixed-methods approach that emphasized qualitative methods over quantitative ones, with integration during the analysis stage. Specifically, we investigated friendships among 31 assisted living residents with moderate to advanced dementia. The Friendship Study was based on the growing literature on social interactions in dementia settings. ‘Social interactions’ describe communicating, verbally and non-verbally, at least once with another person while ‘friendship’ suggests a deeper, more meaningful connection that may include reciprocity, intimacy, and shared trust. From our qualitative analyses, we found that friendships among residents were characterized by voluntary participation and accommodation in conversation, and recognition of the uniqueness of the other. Further quantitative results revealed no correlation between memory test scores, or demographic characteristics (except gender) and friendship dyads identified by staff. Staff’s perceptions of residents’ friendships were not supported through observation. This paper will explore the variety of qualitative methods employed (e.g., ethnographic observation, semi-structured interviews, transcription, coding) and outline how these data were collected, managed, and synthesized. Best practices will be provided.

